# 566. Burn Injury Alters Peripheral Blood Mononuclear Cell Bioenergetics

**DOI:** 10.1093/jbcr/irag033.306

**Published:** 2026-04-06

**Authors:** Meagan S Kingren, Lillie D Treas, Mary Barre, Madeline Crisp, Abby Barlow, Jaycelyn Hall, Eva Diaz, David D Church, Elisabet Borsheim, Miranda L Yelvington, Esther H Teo, Craig Porter

**Affiliations:** University of Arkansas for Medical Sciences, Little Rock, AR; Arkansas Children's Research Institute, Little Rock, AR; Arkansas Children's Research Institute, Little Rock, AR; Arkansas Children's Research Institute, Little Rock, AR; Arkansas Children's Research Institute, Little Rock, AR; University of Arkansas for Medical Sciences, Little Rock, AR; University of Arkansas for Medical Sciences, Little Rock, AR; University of Arkansas for Medical Sciences, Little Rock, AR; Arkansas Children's Burn Program, Little Rock, AR; Arkansas Children's Burn Program, Little Rock, AR; University of Arkansas for Medical Sciences, Little Rock, AR; University of Arkansas for Medical Sciences, Little Rock, AR

## Abstract

**Introduction:**

Hypermetabolism, an increase in whole body oxygen consumption, is a hallmark of the metabolic stress response to burns. Increased cellular oxygen consumption has been described in several organs post burn, yet it remains unclear how burn injury impacts mitochondrial respiration in peripheral blood mononuclear cells (PBMCs).

**Methods:**

Children and adults admitted for acute burn injury and unburned individuals were recruited and studied. Blood samples were collected from patients and controls. A complete blood count with white cell differential was performed on blood samples. Thereafter, PBMCs were isolated by differential centrifugation. PBMC bioenergetics were assayed by high-resolution respirometry and metabolic flux analysis.

**Results:**

Complete blood count with differential analysis revealed burn patients had lower red blood cell count, hemoglobin content, and hematocrit levels (*p*<.001 for all). In contrast, burn led to a greater white blood cell (WBC) count (~2-fold, *p*<.001) and platelet number (*p*<.05). WBC differential analysis revealed quantitative rather than qualitative changes in response to burns, where absolute concentrations of monocytes (*p*<.001) and neutrophils (*p*<.01) were elevated in burn patients. Respiratory capacity and respiration linked to ATP production in intact PBMCs were both ~2-fold higher in burn patients compared to controls (*p*<.01). Similarly, ATP production rate was elevated in adherent PBMCs, which was attributable to increased glycolytic ATP production (*p*<.01). In with this, basal glycolysis and glycolytic flux were also greater in adherent PBMCs from burn patients compared to controls (*p*<.01).

**Conclusions:**

Burn injury results in increased PBMC concentration and altered bioenergetics. PBMCs from burn patients are capable of greater ATP production per cell, potentially due to enhanced glycolytic flux.

**Applicability of Research to Practice:**

Identifying the role of PBMC bioenergetics in the broader hypermetabolic response to burns may lead to potential therapeutics aimed at limiting chronic hypermetabolism and immune dysfunction.

**Funding for the study:**

NIGMS; USDA-ARS; Arkansas Bioscience Institute.

